# The Effectiveness of Plasma Skin Regeneration (PSR) in the Treatment of Chronic Cleft Lip Scars in an Adult Syrian Sample: A Cohort Study on a Novel Technique

**DOI:** 10.7759/cureus.32989

**Published:** 2022-12-27

**Authors:** Abdalmoeen Almohammad, Basel Brad, Amer M Owayda

**Affiliations:** 1 Maxillofacial Surgery Department, Damascus University, Damascus, SYR; 2 Orthodontics, Faculty of Dentistry, University of Hama, Damascus, SYR

**Keywords:** scar management, skin treatment, scar, plasma skin regeneration technique (psr), cleft lip

## Abstract

Objectives

The current study aimed to evaluate the effectiveness of plasma skin regeneration (PSR) in the treatment of cleft lip scars in cleft lip patients.

Materials and methods

Twenty patients, 10 females and 10 males, with a mean age of 19 years and who had a cleft lip scar, were included in the current study. All patients were treated with a plasma skin regeneration pen device in one treatment session. The thickness, relief, and pliability of the scars were assessed by external observers using a 10-point numeric rating scale (NRS).

Results

The thickness, relief, and pliability of the scar were significantly improved according to the observers’ opinions (51.67%, 50.25%, and 46.33%, respectively).

Conclusions

Within the limits of this study, the PSR appeared to be safe and effective for treating cleft lip scars with minimal complications.

## Introduction

A cleft lip is a congenital development defect during the embryonic development period, resulting from the lack of connection of the upper lip tissues [1]. The cleft lip is a physical separation between the two sides of the upper lip. In the case of a cleft lip at the level of skin, muscle, and mucosa, the cleft affects the height of the upper lip [2]. A cleft lip affects the appearance, function of the face, and social-psychological development of the child negatively [3]. Usually, the surgical correction of clefts is performed between the ages of two and six months, and it is the only method of treatment for primary cleft lip. The surgical techniques used to repair lip clefts vary, but the formation of a scar is inevitable after the surgical procedure, regardless of the type of surgical technique used [4]. Many studies have indicated that there are many ways to prevent the shrinkage and enlargement of scar tissue as well as ensure proper tissue healing [5]. The treatment of cleft lip scars involves both non-surgical and surgical methods. The nonsurgical methods include mechanical intervention [6], laser treatment [7], and the usage of chemical substances [8] (e.g., silicone-based products, botulinum toxin injections into muscles, and laser therapy).

Plasma skin regeneration (PSR) is the fourth state of matter, and it is a new technique of resurfacing that creates a thermal effect on the skin using plasma energy [9]. The plasma is emitted from the distal end of the device and is directed to the scar to be treated. Plasma energy differs from the laser in that it does not depend on chromophores and does not evaporate tissues [9]. On the other hand, it leaves a layer of intact, desiccated epidermis that acts as a natural biologic dressing and promotes wound healing and rapid recovery [9]. An in vivo study showed that plasma energy can consistently lead to thermal injury in the papillary dermis, resulting in collagen remodeling without permanent chromosomal or histopathological abnormalities [10]. The histological studies performed on plasma energy confirmed continued collagen production and progressive skin rejuvenation beyond one year after treatment [11]. One of the most important advantages of this technique is that it does not leave an open wound after application and promotes the quick healing of the epidermal layer, which does not exceed seven days. According to the non-fractionated energy of PSR (effectively full-field), there is a possibility of using it in skin regeneration for different types of skin deformities [7, 12]. Plasma energy has been used as an alternative to ablative and fractional resurfacing lasers, with the benefits of a better safety profile and lower cost [13, 14]. Several studies have confirmed that PSR removes benign skin lesions with similar effectiveness and a lower complication rate compared to the carbon dioxide laser in the treatment of traumatic scars [7,15] and in the treatment of scars of the upper lip with focal hypertrophy at the peaks caused by the emerging sutures [16].

The current study aimed to evaluate the effectiveness of this novel method in the field of maxillofacial surgery, especially in patients who have a cleft lip, due to its advantages and the few reported side effects.

## Materials and methods

Trial design

This was a cohort study. The current trial was achieved at the Department of Maxillofacial Surgery at the Faculty of Dentistry, Damascus University, Syria, between September 2019 and January 2022. Ethical approval was obtained from the Local Ethics Research Committee at the University of Damascus Dental School (Reference Number: 297325082019-DEN).

Participants and eligibility criteria

Twenty adult Syrian patients aged 17-23 years (10 males and 10 females, a mean age of 19 years) were enrolled in the current study.

Inclusion criteria: Patients who had a chronic cleft lip scar of type III or IV according to Fitzpatrick skin classification [13]. The age of the scar was greater than five years in all patients.

Exclusion criteria: Patients with pregnancy and lactation, a history of keloid formation, previous laser treatment, a history of collagen vascular disease, a history of dermal fillers, and an inability to avoid significant sun exposure during the follow-up period were excluded.

Informed consent was obtained from all patients before any procedure was applied.

Sample size calculation

A sample size calculation was done using Minitab® 18.1 (INC, Pennsylvania, USA) according to the following assumptions: The minimal clinical difference to be detected was 2 degrees on the Numeric Rating Scale (NRS); the standard deviation was determined to be two as well; the level of significance (alpha) was set at 0.05 with a power of 85%. According to the calculation, the required sample size was 20 patients.

The plasma skin regeneration procedure (PSR)

**Figure 1:**
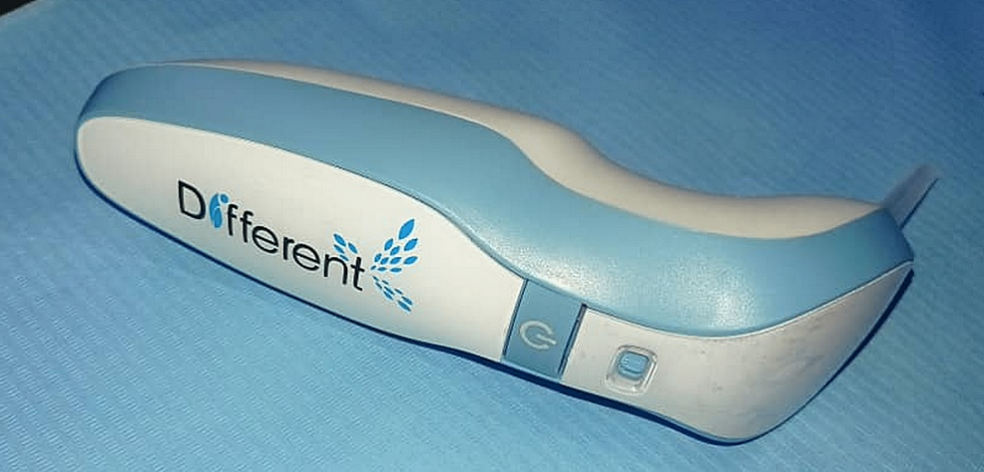
The plasma skin regeneration device (Plasma Pen, Maglev, Germany)

The patients were treated with plasma skin regeneration in one stage, using the PSR device (Plasma Pen, Maglev, Germany) (Figure 1). The applied energy ranged from 3-4 joules (which is safe according to the US Food and Drug Administration [9]) for each scar.

First, the area was disinfected with a povidone-iodine solution. Before the PSR treatment, patients were given cephalexin 500 mg twice daily (BID) and acyclovir 400 mg three times a day (TID) for bacterial and viral prophylaxis, respectively. A topical 4% lidocaine gel (lidocaine 4% gel, Ibn Zohr, Damascus, Syria) was applied to the treatment site, followed by an injection of 2% lidocaine (lidocaine 2%, Ibn Zohr, Damascus, Syria).

After anesthesia, the skin was disinfected with hexamidine (trans hexamidine, Al-Asi, Hama, Syria), followed by saline irrigation.

**Figure 2:**
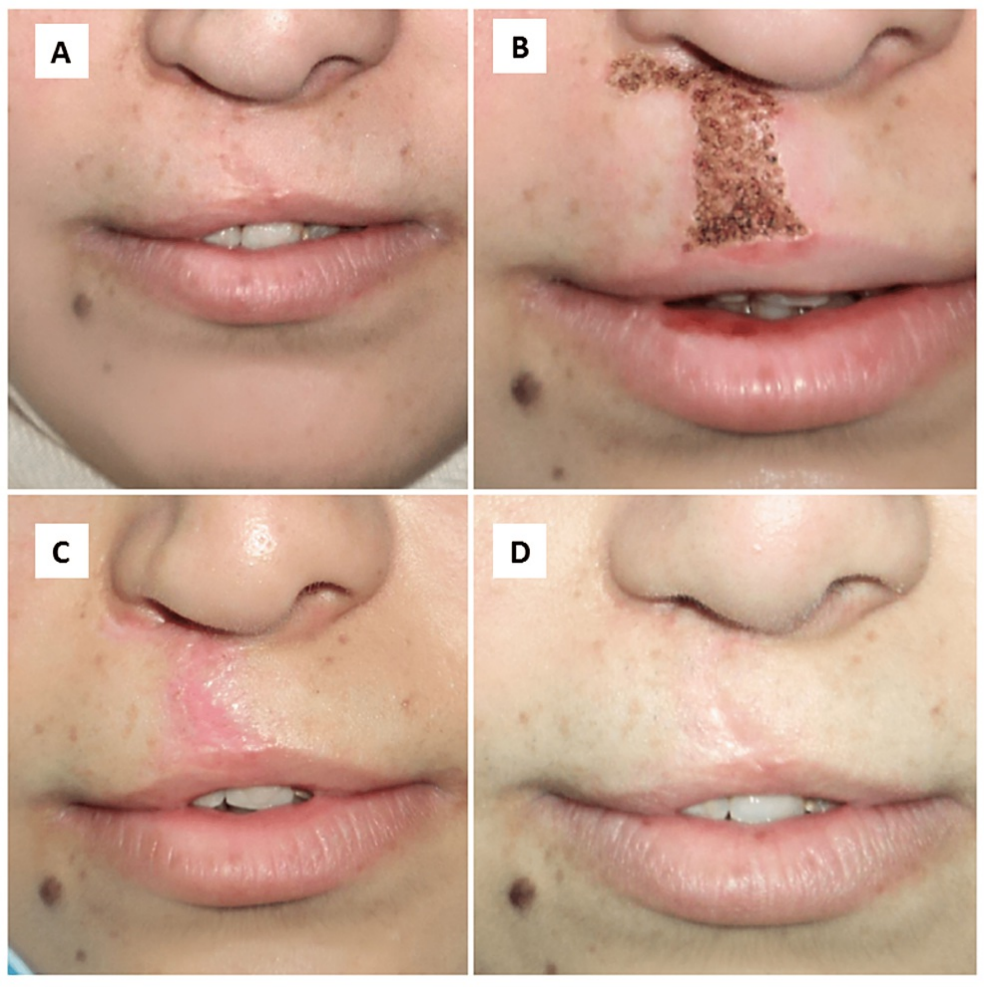
Clinical case A: before treatment; B: immediately after PSR treatment; C: after one week of PSR treatment; D: after six months of PSR treatment.

A single pass of high-energy plasma was delivered to the scar and the surrounding normal skin for 15 minutes. The tip of the pen did not touch the surface of the treated skin. All patients were asked to use a moisturizing cream and not itch the treatment site for a week. Figure 2 shows one of the clinical cases treated with PSR during this trial.

Postoperative care consists of an open dressing with petrolatum-based ointment, which is applied three to four times daily, along with gentle cleansing with a non-detergent-based cleanser. Sunscreens and makeup are generally safe to use following desquamation by postoperative day seven following treatment.

Outcomes assessment

**Figure 3:**
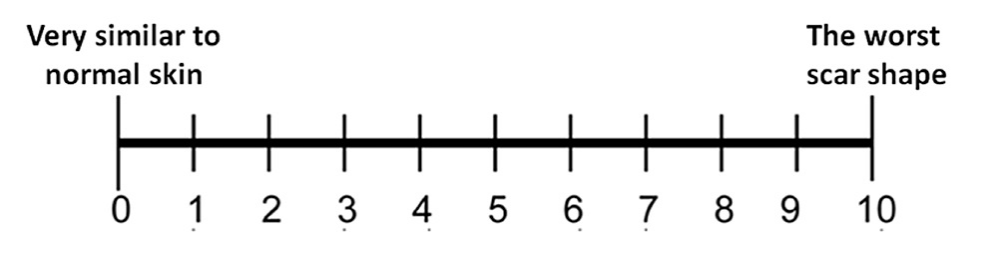
The numeric rating scale (NRS)

The thickness, relief, and pliability of the cleft scar were assessed before treatment and six months after the treatment by three external observers from the Oral and Maxillofacial Surgery Residents (the residents were master's and PhD-postgraduate students in the department of oral and maxillofacial surgery at Damascus University, and they were randomly selected from the oral and maxillofacial surgery department committee). The observers had no conflict of interest either. They used a 10-point numeric rating scale (NRS), with one referring to an appearance very similar to normal skin and 10 referring to the worst scar shape (Figure 3). The mean value of the three external observers’ readings was considered for the statistical analysis. Thickness refers to the distance between the surface of the scar and the adjacent skin surface. Relief refers to the extent to which surface irregularities are present. Pliability is measured by the flexibility of the scar between the index finger and thumb.

Statistical analysis

All statistical analyses were performed using Statistical Package for Social Sciences (SPSS) software (version 20; IBM, Armonk, New York, USA). The Shapiro-Wilk test was used to assess the shape of the data distribution and its normality. A Wilcoxon signed-rank test was used to detect significant changes in the scar properties after PSR treatment.

## Results

The percentage of improvement for the thickness, relief, and pliability of the scar was assessed according to the opinions of the observers using the following equation:

The Improvement Percentage = (the amount of improvement after treatment / pre-operative rate) * 100.

The thickness, relief, and pliability of the scar were improved according to the observers’ opinions (51.67%, 50.25%, and 46.33%, respectively) (Table 1).

**Table 1:** The descriptive values in both the thickness and elasticity of the skin The amount of improvement: ( postoperative rate value - preoperative rate value)

Studied item	Before mean (SD)	After mean (SD)	The amount of improvement mean (SD)	improvement percentage mean (SD)
Thickness	4.75 (0.64)	2.30 (0.65)	-2.45 (0.60)	-51.67 (11.64)
Relief	5.15 (0.93)	2.55 (0.88)	-2.60 (0.94)	-50.25 (15.53)
Pliability	4.35 (0.58)	2.35 (0.59)	-2.00 (0.46)	-46.33 (10.41)

The thickness, relief, and pliability significantly improved after the PSR, according to the outer observer’s reports (p<0.05) (Table 2).

**Table 2:** Comparison of the thickness, relief, and pliability of the lip scar before and after the plasma skin regeneration operation *: p<0.05 significant difference; #: the Wilcoxon signed-ranks test was applied

Variable	Time	N	Mean ±SD	p-value #
Thickness	Before	20	4.75 (0.64)	<0.001*
	After	20	2.30 (0.65)	
Relief	Before	20	5.15 (0.93)	<0.001*
	After	20	2.55 (0.88)	
Pliability	Before	20	4.35 (0.58)	<0.001*
	After	20	2.35 (0.59)	

## Discussion

The current study assessed the PSR as a modality for enhancing the scar of the cleft lip. Carbon dioxide lasers are more frequently used in the field of facial scar treatment. Unfortunately, carbon dioxide lasers may cause damage to the skin, and side effects include transient or prolonged erythema, temporary and permanent hypopigmentation, hyperpigmentation infection, and scarring [17, 18].

Plasma skin regeneration technology has been evaluated in several medical fields, including complete regeneration of the facial skin at low energy [19]. It was evaluated in the treatment of wrinkles around the eyes and around the mouth [9]. This technique was evaluated in the treatment of traumatic scars in Asian patients [15]. Recently, a case report was published on the treatment of a scar on the upper lip with focal hypertrophy at the peaks caused by the emerging sutures, following surgical excision of skin cancer using plasma energy technology, and it showed a significant improvement after follow-up [16].

During the first ten days of PSR, which includes the superficial skin layers and uses a high energy rate (3-4 joules), the regeneration reaches the upper papillary layer. Continuous collagen production starts from the beginning of the treatment and lasts for a year around the area of thermal damage resulting from the treatment. It differs from the carbon dioxide laser in two main points: the tissue surface contraction, as a result of dermal collagen thermal denaturation, and the thermal disruption of solar elastosis; and the activation of fibroblasts stimulate a wound healing cascade necessary for neocollagenesis and the reduction of solar elastosis [7, 9, 11].

In the current study, high energy (3-4 joules) was used to treat the scars. This energy can produce a high-frequency thermal effect on the surface of the skin [9].

The results of the current report showed that PSR improved the thickness, relief, and pliability of the scar. These results can be explained by the polished surface after treatment as a result of the denaturation of collagen fibers due to carbonizing the spot, the reorganization of collagen and elastin fibers into tissue on the surface of the dermal-epidermal junction, and regeneration processes that include the epidermal layers and the upper layers of the dermis in cases of high energy treatment [9].

This thermal effect caused by plasma energy helps to improve the characteristics of the scar and the skin adjacent to it in terms of pliability, thickness, and relief. These results agreed with the study of Kono et al., who evaluated the traumatic scars and found an improvement in the superficial and medium-depth scars of up to 50%, while deep scars showed resistance to treatment [15].

The results of the current study can be linked to the treatment of wrinkles. The same findings were observed in the study of Foster et al., who assessed the wrinkles around the mouth after the PSR treatment and reported a high improvement rate [9]. These findings can be explained by the continued collagen production around the thermal injury areas, which continues for a year after treatment and whose regularity increases around the dermal-epidermal junction [9], as well as the formed carbon scabs, which have a role in protecting the treatment area as a natural dressing and can explain the lack of complications following treatment from infection and inflammation.

The main limitation of the current study is that the complications of the PSR procedure were not assessed. Another limitation is that the current study did not evaluate symmetry during dynamic aspects of lip movements. Finally, the current study is limited in that it did not study the effect of repeating the application of the procedure (PSR) on its effectiveness.

## Conclusions

Within the limits of the current study, a 15-minute single pass of high-energy plasma can make an average improvement in chronic cleft lip scar thickness, relief, and pliability, according to the opinions of external observers. The PSR can be considered an effective modality of treatment for enhancing the scarring properties of cleft lips in adult Syrian cleft lip patients. 

## References

[1] Silva HP, Arruda TT, Souza KS (2018). Risk factors and comorbidities in Brazilian patients with orofacial clefts. Braz Oral Res.

[2] Clark JM, Skoner JM, Wang TD (2003). Repair of the unilateral cleft lip/nose deformity. Facial Plast Surg.

[3] Chen G, Li MX, Wang HX (2018). Identification of key genes in cleft lip with or without cleft palate regulated by miR-199a-5p. Int J Pediatr Otorhinolaryngol.

[4] de Korte CL, van Hees N, Lopata RG, Weijers G, Katsaros C, Thijssen JM (2009). Quantitative assessment of oral orbicular muscle deformation after cleft lip reconstruction: an ultrasound elastography study. IEEE Trans Med Imaging.

[5] Papathanasiou E, Trotman CA, Scott AR, Van Dyke TE (2017). Current and emerging treatments for postsurgical cleft lip scarring: effectiveness and mechanisms. J Dent Res.

[6] Peng L, Tang S, Li Q (2018). Intense pulsed light and laser treatment regimen improves scar evolution after cleft lip repair surgery. J Cosmet Dermatol.

[7] Fitzpatrick R, Bernstein E, Iyer S, Brown D, Andrews P, Penny K (2008). A histopathologic evaluation of the plasma skin regeneration System (PSR) versus a standard carbon dioxide resurfacing laser in an animal model. Lasers Surg Med.

[8] Chang CS, Wallace CG, Hsiao YC, Chang CJ, Chen PK (2014). Botulinum toxin to improve results in cleft lip repair: a double-blinded, randomized, vehicle-controlled clinical trial. PLoS One.

[9] Foster KW, Moy RL, Fincher EF (2008). Advances in plasma skin regeneration. J Cosmet Dermatol.

[10] Tremblay JF, Moy R (2004). Treatment of post-auricular skin using a novel plasma resurfacing system: an in vivo clinical and histologic study. Lasers Surg Med.

[11] Rhytec Inc. (2008). Rhytec True Regenerative Science. https://www.accessdata.fda.gov/cdrh_docs/pdf7/K073111.pdf.

[12] Kilmer S, Semchyshyn N, Shah G, Fitzpatrick R (2007). A pilot study on the use of a plasma skin regeneration device (Portrait PSR3) in full facial rejuvenation procedures. Lasers Med Sci.

[13] Fitzpatrick R, Geronemus R, Kim K, Brown D, Bernstein E (2004). Plasmakinetic skin rejuvenation on peri-oral rhytides. Lasers Surg Med.

[14] Potter M, Harrison R, Ramsden A, Penny K, Andrews P, Gault D (2003). A randomized control trial comparing plasma skin resurfacing (PSR) with carbon dioxide laser in the treatment of benign skin lesions. Lasers Med Sci.

[15] Kono T, Groff WF, Sakurai H, Yamaki T, Soejima K, Nozaki M (2009). Treatment of traumatic scars using plasma skin regeneration (PSR) system. Lasers Surg Med.

[16] Baroni A (2020). Preliminary assessment for postsurgical scar treatment with the novel low-energy plasma skin regeneration technique. Indian J Dermatol.

[17] Nanni CA, Alster TS (1998). Complications of carbon dioxide laser resurfacing. An evaluation of 500 patients. Dermatol Surg.

[18] Walia S, Alster TS (1999). Prolonged clinical and histologic effects from CO2 laser resurfacing of atrophic acne scars. Dermatol Surg.

[19] Bogle MA, Arndt KA, Dover JS (2007). Evaluation of plasma skin regeneration technology in low-energy full-facial rejuvenation. Arch Dermatol.

